# Diversity parameter calculation from SSR data with varying and higher ploidy levels: an example on pear (*Pyrus* ssp.) data

**DOI:** 10.1186/s13104-026-07953-w

**Published:** 2026-08-10

**Authors:** Lea Broschewitz, Stefanie Reim, Henryk Flachowsky, Monika Höfer

**Affiliations:** Julius Kühn Institute (JKI), Federal Research Centre for Cultivated Plants, Institute for Breeding Research on Fruit Crops, Pillnitzer Platz 3a, 01326 Dresden-Pillnitz, Germany

**Keywords:** Genetic resources, Molecular markers, Genetic diversity, Ploidy, Allele variants

## Abstract

**Objective:**

SSR markers are used for a myriad of genetic analyses, such as genotyping, parentage analysis or genetic structure, due to their high polymorphism, co-dominance and genomic ubiquity. Most downstream analysis tools usually require a diploid dataset but some plant species may have higher ploidy levels or there may be genotypes within the species that differ in ploidy level. Additionally, SSR markers can show multi-locus behaviour and are prone to genotyping errors. Consequently, existing analysis tools often do not meet the requirements of plant datasets. Nevertheless, accurately estimating genetic diversity parameters remains essential, even for polyploid datasets. This emphasizes the importance of adapting such datasets appropriately to enable subsequent analysis while preserving their biological validity.

**Results:**

This study examines an example dataset of pear cultivars to evaluate how genetic diversity parameters are affected by data adjustments. The original dataset, which includes numerous markers with more than two alleles, was compared with three modified subsets in which additional alleles (more than two) and selected markers were excluded. The results show that genetic diversity statistics remain largely consistent across datasets. The script used in this work thus provides a robust basis for making dataset adjustments before further analyses.

**Supplementary Information:**

The online version contains supplementary material available at 10.1186/s13104-026-07953-w.

## Introduction

Simple sequence repeat (SSR) markers, also called microsatellites, are used for a wide range of analyses due to their highly polymorphic, co-dominant and genomic ubiquity [1–4]. Such analyses include DNA fingerprinting, parentage analysis and studies of population genetics [5–8]. Most downstream analysis tools for SSRs are designed for diploid organisms, e.g. CERVUS [9, 10], GenAlex [11], or homogenous polyploid populations, e.g. STRUCTURE [12, 13]. However, higher ploidy levels are common in plants, which can make the analysis with the common tools more difficult. The R package polysat offers analysis of mixed polyploid population but standard genetic diversity parameters are not calculated [14]. Additionally, SSR markers can produce more than the anticipated two allele variants for diploid individuals when multiple binding sites are present in the genome. This is known as a multi-locus SSR marker. In plant genomes, this can happen due to segmental duplication as well as syntenic or paralogous regions [15]. Multi-locus markers can be a powerful tool for genotyping, as they retain more information. However, the allele variants must be clearly defined so that they can be assigned to their genomic origin. Another reason why SSRs could express more allele variants than expected is their susceptibility to genotype errors, such as allele calling errors [16, 17]. These factors can present obstacles to the downstream analysis of SSR data, because the genotype profile may differ from the required input data format. One approach would be to exclude markers showing multi-locus behaviour from the analysis. However, multi-locus behaviour often only occurs in certain genotypes. This would result in the loss of information for the entire dataset and reduced statistical power [16].

In this study, we analysed a pear dataset in which diploid individuals exhibit more allele variants than expected [18]. To facilitate downstream analysis, we examine both the original data and adjusted subsets that comply with the input requirements of common bioinformatic tools. For this purpose, we developed a script that assesses the suitability of the original and modified datasets by calculating established genetic diversity parameters [19]. With this work, we aim to raise awareness for this common but often unaddressed issue in SSR data analysis and to demonstrate an approach for evaluating dataset adjustment and their impact on genetic diversity statistics.

## Main text

### Methods

For this exemplary analysis, we used the pear cultivar (*Pyrus* ssp.) dataset from the German Fruit Genebank (GFG), which is available through the OpenAgrar repository [18]. This dataset comprises the genetic data for 421 pear varieties, which were molecularly analysed using 17 SSRs recommended by the European Cooperative Programme for Plant Genetic Resources (ECPGR, www.ecpgr.org) and pomologically tested for trueness-to-type [18, 20, 21]. The generation of the data set is described in detail by Broschewitz et al. (2026) [22]. Namely, the used SSR markers are CH04c07, CH02b10, CH05c06, EMPc117, GD96, EMPc11, GD142, CH01d09, GD147, CH01d08, CH03g07, CH01f07a, CH-Vf1, NZ05g8, CH04e03, CH05a02, CH03d12 [21]. To calculate the genetic diversity parameters commonly used to evaluate SSR datasets, we developed an R script named “*SSR_DivParCal*” [19, 23]. The R script uses the packages readxl [24], adegenet [25, 26] and openxlsx [27]. In specific, the calculated diversity parameter were number of alleles *N*_*a*_, effective number of alleles *N*_*e*_ [28], observed heterozygosity *H*_*obs*_, expected heterozygosity *H*_*exp*_ [29], polymorphic information content *PIC* [30, 31], probability of identity for unrelated individuals *PID* [32] and probability of identity for siblings *PID*_*sib*_ [33]. The R script “*SSR_DivParCal*” can deal with varying and higher ploidy levels in the provided dataset. In this approach, missing data is excluded from the calculation of allele frequencies. The diversity parameters were calculated for the original dataset (‘original’) retaining each allele variant as well as three subsets: (I) markers CH01d09 and CH05a02 removed (‘rem2markers’), (II) forced diploid dataset (‘force2x’) where only the first two allele variants of each marker are retained, and (III) the combination of rem2markers and force2x (‘rem2markers_force2x’). For ‘rem2markers’, CH01d09 and CH05a02 were removed as both markers showed up to six allele variants for several genotypes/cultivars of the used dataset. Furthermore, CH01d09 showed more than two allele variants in several reference genotypes of the current dataset and CH05a02 showed three or four allele variants for all reference genotypes in the current dataset and the ECPGR reference data (Supplemental Table 1) [21]. The subsets ‘forced2x’ and ‘rem2markers_forced2x’ were analysed with default settings in CERVUS and GenAlex to validate the results (Supplemental Tables 3 & 4). For comparison, PIC values for all datasets were calculated with R package polysat (Supplemental Table 5). Wilcoxon signed rank test was performed between the original data and the subsets for *H*_*exp*_, *PIC*, *PID* and *PID*_*sib*_.

### Results

For some of the pear cultivars, the ploidy levels were previously determined by flow cytometry (92 diploid, 17 triploid, 312 not tested) [18], although pear cultivars are generally assumed to be predominantly diploid [34]. Manual inspection of the genotypes revealed that many individuals display more allele variants than expected based on the ploidy level, i.e. two allele variants for diploid cultivars. This was also observed when comparing the current data for the ECPGR reference genotypes with the information from the literature, i.e. for marker CH05a02 (Supplemental Table 1) [21].

The mean results of the genetic diversity parameters of the four datasets (‘original’, ‘rem2markers’, ‘force2x’ and ‘rem2markers_force2x’) can be found in Table 1. The marker-specific results of the four datasets are stored in Supplemental Table 2. The genetic diversity represented by Ø *H*_*exp*_ is highest in the ‘original’ dataset (Ø *H*_*exp*_ = 0.83) and lowest in the ‘rem2markers_force2x’ dataset (Ø *H*_*exp*_ = 0.81). The highest variation of values between the tested datasets is for the genetic diversity parameter Ø *N*_*a*_ and Ø *N*_*e*_. For the remaining diversity parameter Ø *H*_*obs*_, Ø *PIC*, Ø *PID* and Ø *PID*_*sib*_, the values show a variation of 0.3 or less. A Wilcoxon signed rank test between the original data and the subsets for *H*_*exp*_, *PIC*, *PID* and *PID*_*sib*_.reveals that the ‘force2x’ and ‘rem2markers_force2x’ dataset significantly differ from the ‘original’ dataset (data not shown).

**Table 1:** Mean genetic diversity parameters of four testing datasets (original dataset and 3 modified datasets)

Datasets	Original	rem2markers	force2x	rem2markers_force2x
Parameters				
Total individuals	421	421	421	421
Total markers	17	15	17	15
Ø individuals assessed	414.5	413.7	414.5	413.7
Ø *N*_*a*_	20.82	20.73	19.53	20
Ø *N*_*e*_	7.06	6.60	6.44	6.43
Ø *H*_*obs*_	0.77	0.74	0.77	0.74
Ø *H*_*exp*_	0.83	0.82	0.82	0.81
Ø *PIC*	0.81	0.80	0.80	0.80
Ø *PID*	0.06	0.06	0.06	0.06
Ø *PID*_*sib*_	0.35	0.36	0.36	0.36

The modified datasets ‘forced2x’ and ‘rem2markers_forced2x’ were additionally analysed using the CERVUS and GenAlex software (Supplemental Tables 3 & 4). Using both software packages, the following parameters were compared with the results obtained from the “*SSR_DivParCal*” script: Ø individuals assessed, Ø *N*_*a*_, Ø *H*_*exp*_ and Ø *H*_*obs*_. In addition, CERVUS was used to calculate Ø *PIC* and GenAlex was used to calculate Ø *N*_*e*_. The metrics calculated by each program were identical to the results of the “*SSR_DivParCal*” script (see Table 1, Supplemental Tables 2, 3 & 4). All datasets were processed by polysat to calculate PIC (Supplemental Table 5).

### Discussion

The initial pear cultivar dataset from the GFG was not suitable for directly calculating genetic fingerprint diversity parameters, as it exhibited more allele variants than expected [18]. Most bioinformatic tools used to estimate genetic diversity require marker information of diploid individuals in a dataset. Although pear is predominantly diploid, some triploid individuals were reported [18]. Additionally, several marker loci showed allele profiles with up to six unique allele variants. Differences in ploidy level can generally not be processed by most bioinformatics tool, which are mainly based on diploid mammalian species, e.g. CERVUS [9, 10]. Many fruit species are reported to show varying levels of ploidy: apple [35, 36], citrus [37], sour cherry [38], grape vine [39] and kiwi [40], including pear [41]. One tool that can handle varying degrees of ploidy is the R package polysat [14]. While polysat is powerful when evaluating different populations, i.e. with F_ST_ values [29], many more basic parameters are omitted. The “*SSR_DivParCal*” script calculates the most common diversity parameters based on equation from literature, retaining all allele variants and without requiring explicit knowledge of the ploidy level. In this work, we demonstrate the feasibility and applicability of this script and show that it can serve for a preliminary analysis to estimate datasets, even though distinct analyses i.e. with CERVUS or STRUCTURE, are not replaceable.

The range of Ø *H*_*exp*_ across the test datasets is very small, with a maximum range of 0.2. The Ø *H*_*exp*_ values in this study are comparable to other pear germplasm collections, e.g. from Italy [42], Spain [43, 44], Tunisia [45, 46], Portugal [47], etc. As expected the ‘original’ dataset showed the highest Ø *H*_*exp*_ as here all allele variants and markers are retained (see Table 1). Statistically, the marker-specific *H*_*exp*_ differed significantly (*p* < 0.001) between ‘original’ and ‘force2x’ dataset (data not shown). Interestingly, the Ø *H*_*obs*_ is identical between the ‘original’ and ‘forced2x’ dataset, indicating that dataset diversity does not appear to be substantially affected by the removal of excess alleles. Based on the genetic diversity parameters, the removal of alleles as in the ‘forced2x’ dataset has the highest impact on the parameters itself.

The functionality and validity of the “*SSR_DivParCal*” script was estimated using suitable test datasets by comparing its results with those generated by commonly tools for analyzing genetic diversity, namely CERVUS and GenAlex. The outcomes were identical, proving the functionality of the script at a level comparable to the commonly used and available alternatives. R package polysat was used to calculate PIC across all datasets and showed no significantly different (*p* < 0.001) outcomes.

As mentioned before, the number of allele variants is determined by the genotype and ploidy level. The occurrence of more than two alleles can also result from multi-locus behaviours of certain markers. This may be caused by syntenic blocks located on different pear chromosomes or by technical issues during marker detection. Pear is known to has a high degree of self-synteny with collinear gene blocks [15]. SSR markers may therefore bind to these syntenic regions, resulting in more than two allele variants even in diploid species. Multi-locus behaviour is already reported by the original ECPGR study with the reference genotypes for markers CH01d08, CH-Vf1 and CH05a02 [21]. In the original pear data, CH05a02 and CH01d09 showed up to 6 different allele variants per genotype and were therefore subsequently completely excluded in the present analysis (Supplemental Table 1) [18]. Of all ECPGR marker used in our study, CH05a02 was described as multi-locus marker on the HiDRAS website, which is in line with our results [21, 48]. For the remaining markers the information was partially missing and all HiDRAS information for the selected markers referred to apple rather than pear.

Technical issues can also lead to the detection of additional alleles. For example, stutter bands can mistakenly be detected as individual allele variants during the chromatogram analysis [7, 49]. These stutter bands are common in dinucleotide SSRs, as the SSR markers in this study. Using SSR markers with tri-, tetra- or pentanucleotide repeat patterns can alleviate the formation of stutter bands and increase the quality of the data by reducing miscalled allele variants [50].

It should be emphasized once again that, for none of the markers in the dataset, a purely diploid allele pattern with only two allele variants could be obtained. The literature does not always clearly describe how researchers tackle this problem e.g. whether they restrict analysis to cultivars that display only diploid allele patterns or removing extra alleles, as one in this study. It is important to note that with this approach may skew allele frequencies and rare alleles may be lost.

Evaluating different datasets and subsets with the “*SSR_DivParCal”* script prior to final analyses helps to ensure data suitability and increases transparency in data handling [19]. In case of the pear example, proceeding with ‘rem2markers_forced2x’ dataset appears to be a reasonable approach for further analysis, i.e. genetic structure.

### Limitations

- For parentage analysis and identity by descent analysis, retaining all alleles is crucial to maintain the analytic power. Differences in ploidy pose a particular challenge, as null alleles are more likely to be missed in triploid individuals, and it becomes impossible to determine which allele is duplicated when only two variants are detected.
- Rare allele variants may be removed by data modification and therefore not included in the downstream analysis. While this may influence the results, the use of the “*SSR_DivParCal”* script and together with comparison of the different datasets can give an indication whether such losses are problematic, in which case alternative approaches should be considered.
- This is an example for a specific dataset. For other datasets, the exclusion of allele variants may have stronger negative effects. Nonetheless, this approach provides a means to evaluate how adjustments to a dataset influence genetic diversity parameters when such modifications are necessary.

## Supplementary Information

Supplementary Material 1.

## Data Availability

The data analysed during the current study are filtered from a previously published dataset freely available in the OpenAgrar repository: https://doi.org/10.5073/20250814-135212-0 [16]. Extensive description of the data sourcing is provided by the respective data descriptor: https://doi.org/10.1038/s41597-026-07010-y [22].

## Abbreviations

GFG: German Fruit Genebank
PUNQ: Pyrus Unique genotype code
SSR: Simple sequence repeat

## References

[1] Weber JL, May PE. Abundant class of human DNA polymorphisms which can be typed using the polymerase chain reaction. Am J Hum Genet. 1989;44(3):388–96. 2916582 PMC1715443

[2] Gupta PK, Balyan HS, Sharma PC, Ramesh B. Microsatellites in plants: A new class of molecular markers. Curr Sci. 1996;70(1):45–54.

[3] Bhargava A, Fuentes FF. Mutational dynamics of microsatellites. Mol Biotechnol. 2010;44(3):250–66. 10.1007/s12033-009-9230-4. 20012711 10.1007/s12033-009-9230-4

[4] Oliveira EJ, Pádua JG, Zucchi MI, Vencovsky R, Vieira MLC. Origin, evolution and genome distribution of microsatellites. Genet Mol Biol. 2006;29:294–307. 10.1590/S1415-47572006000200018.

[5] Schlötterer C, Pemberton J. The use of microsatellites for genetic analysis of natural populations. In: Schierwater B, Streit B, Wagner GP, De Salle R, editors. Molecular ecology and evolution: approaches and applications. Basel: Springer; 1994. p. 203–14. 10.1007/978-3-0348-7527-1_11. 10.1007/978-3-0348-7527-1_11 7994107

[6] Azizi MMF, Lau HY, Abu-Bakar N. Integration of advanced technologies for plant variety and cultivar identification. J Biosci. 2021;46(4):91. 10.1007/s12038-021-00214-x. 34544910

[7] Zalapa JE, Cuevas H, Zhu H, Steffan S, Senalik D, Zeldin E, et al. Using next-generation sequencing approaches to isolate simple sequence repeat (SSR) loci in the plant sciences. Am J Bot. 2012;99(2):193–208. 10.3732/ajb.1100394. 22186186 10.3732/ajb.1100394

[8] Putman AI, Carbone I. Challenges in analysis and interpretation of microsatellite data for population genetic studies. Ecol Evol. 2014;4(22):4399–428. 10.1002/ece3.1305. 25540699 10.1002/ece3.1305 PMC4267876

[9] Marshall TC, Slate J, Kruuk LEB, Pemberton JM. Statistical confidence for likelihood-based paternity inference in natural populations. Mol Ecol. 1998;7(5):639–55. 10.1046/j.1365-294x.1998.00374.x. 9633105 10.1046/j.1365-294x.1998.00374.x

[10] Kalinowski ST, Taper ML, Marshall TC. Revising how the computer program cervus accommodates genotyping error increases success in paternity assignment. Mol Ecol. 2007;16(5):1099–106. 10.1111/j.1365-294X.2007.03089.x. 17305863 10.1111/j.1365-294X.2007.03089.x

[11] Peakall R, Smouse PE. GenAlEx 6.5: genetic analysis in Excel. Population genetic software for teaching and research—an update. Bioinformatics. 2012;28(19):2537–9. 10.1093/bioinformatics/bts460. 22820204 10.1093/bioinformatics/bts460 PMC3463245

[12] Pritchard JK, Stephens M, Donnelly P. Inference of population structure using multilocus genotype data. Genetics. 2000;155(2):945–59. 10.1093/genetics/155.2.945. 10835412 10.1093/genetics/155.2.945 PMC1461096

[13] Falush D, Stephens M, Pritchard JK Inference of population structure using multilocus genotype data: linked loci and correlated allele frequencies. Genetics. 2003;164(4):1567–87. 10.1093/genetics/164.4.1567. 10.1093/genetics/164.4.1567 PMC1462648 12930761

[14] Clark LV, Jasieniuk M. polysat: an R package for polyploid microsatellite analysis. Mol Ecol Resour. 2011;11(3):562–6. 10.1111/j.1755-0998.2011.02985.x. 21481215 10.1111/j.1755-0998.2011.02985.x

[15] Linsmith G, Rombauts S, Montanari S, Deng CH, Celton JM, Guérif P, et al. Pseudo-chromosome–length genome assembly of a double haploid “Bartlett” pear (*Pyrus communis* L.). Gigascience. 2019;8(12):giz138. 10.1093/gigascience/giz138. 31816089 10.1093/gigascience/giz138 PMC6901071

[16] Selkoe KA, Toonen RJ. Microsatellites for ecologists: a practical guide to using and evaluating microsatellite markers. Ecol Lett. 2006;9(5):615–29. 10.1111/j.1461-0248.2006.00889.x. 16643306 10.1111/j.1461-0248.2006.00889.x

[17] Bonin A, Bellemain E, Bronken Eidesen P, Pompanon F, Brochmann C, Taberlet P. How to track and assess genotyping errors in population genetics studies. Mol Ecol. 2004;13(11):3261–73. 10.1111/j.1365-294X.2004.02346.x. 15487987 10.1111/j.1365-294X.2004.02346.x

[18] Broschewitz L, Schramm B, Bade J, Meyer J, Schulte E, Flachowsky H et al. Microsatellite/SSR dataset: pomological and molecular characterization of pear cultivars (*Pyrus communis*) of the German Fruit Genebank, Version1. 2025. 10.5073/20250814-135212-0.

[19] Broschewitz L. SSR_DivParCal. 2025. 10.5281/zenodo.17510846.

[20] Lateur M, Höfer M, Denancé C, Ikase L, Jiménez CM, Errea P et al. Common ECPGR protocols and tools available for characterization and evaluation of Malus/Pyrus genetic resources (Pome Fruit C&E). Final Rep. Available from: https://www.ecpgr.org/fileadmin/bioversity/publications/pdfs/Pomefruit_activity_report_final.pdf.

[21] Evans KM, Fernández-Fernández F, Govan C. Harmonising fingerprinting protocols to allow comparisons between germplasm collections - *Pyrus*. Acta Hortic. 2009;814:103–6. 10.17660/ActaHortic.2009.814.10.

[22] Broschewitz L, Schramm B, Flachowsky H, Schulte E, Höfer M. Microsatellite/SSR dataset: characterization of pear cultivars of the German Fruit Genebank. Sci Data. 2026;13(1):391. 10.1038/s41597-026-07010-y. 41794880 10.1038/s41597-026-07010-y PMC12993052

[23] R: The R project for statistical computing. Available from: https://www.r-project.org/. Accessed 14 July 2026.

[24] Wickham H, Bryan J, Posit. attribution) P (Copyright holder of all R code and all C code without explicit copyright, code) MK (Author of included R, code) KV (Author of included libxls, readxl: Read Excel Files. 2026. Available from: https://cran.r-project.org/web/packages/readxl/index.html. Accessed 4 July 2026.

[25] Jombart T. Adegenet: a R package for the multivariate analysis of genetic markers. Bioinformatics. 2008;24(11):1403–5. 10.1093/bioinformatics/btn129. 18397895 10.1093/bioinformatics/btn129

[26] Jombart T, Ahmed I. adegenet 1.3-1: new tools for the analysis of genome-wide SNP data. Bioinformatics. 2011;27(21):3070–1. 10.1093/bioinformatics/btr521. 10.1093/bioinformatics/btr521 PMC3198581 21926124

[27] Schauberger P, Walker A, Braglia L, Sturm J, Garbuszus JM, Barbone JM et al. openxlsx: Read, Write and Edit xlsx Files. 2025. Available from: https://cran.r-project.org/web/packages/openxlsx/index.html. Accessed 4 July 2026.

[28] Kimura M, Crow JF. The number of alleles that can be maintained in a finite population. Genetics. 1964;49(4):725–38. 10.1093/genetics/49.4.725. 14156929 10.1093/genetics/49.4.725 PMC1210609

[29] Nei. Analysis of gene diversity in subdivided populations. PNAS. Accessed 4 July 2026. Available from: 10.1073/pnas.70.12.3321.

[30] Botstein D, White RL, Skolnick M, Davis RW. Construction of a genetic linkage map in man using restriction fragment length polymorphisms. Am J Human Genetics. 1980;32:314–31. 6247908 PMC1686077

[31] Serrote CML, Reiniger LRS, Silva KB, Rabaiolli S, Stefanel CM. Determining the polymorphism information content of a molecular marker. Gene. 2020;726:144175. 10.1016/j.gene.2019.144175. 31726084 10.1016/j.gene.2019.144175

[32] Paetkau D, Strobeck C. Microsatellite analysis of genetic variation in black bear populations. Mol Ecol. 1994;3(5):489–95. 10.1111/j.1365-294X.1994.tb00127.x. 7952329 10.1111/j.1365-294x.1994.tb00127.x

[33] Waits LP, Luikart G, Taberlet P. Estimating the probability of identity among genotypes in natural populations: cautions and guidelines. Mol Ecol. 2001;10(1):249–56. 10.1046/j.1365-294X.2001.01185.x. 11251803 10.1046/j.1365-294x.2001.01185.x

[34] Postman J, Bassil N, Bell R. Ploidy of USDA world pear germplasm collection determined by flow cytometry. Acta Hortic. 2015;1094:75–81. 10.17660/ActaHortic.2015.1094.6.

[35] Podwyszyńska M, Marasek-Ciołakowska A. Ploidy, genome size, and cytogenetics of apple. In: Korban SS, editor. The Apple Genome. Cham: Springer International Publishing; 2021. p. 47–71. 10.1007/978-3-030-74682-7_4.

[36] Broschewitz L, Reim S, Flachowsky H, Höfer M. Pomological and molecular characterization of apple cultivars in the German fruit genebank. Plants. 2024;13(19):2699. 10.3390/plants13192699. 39409569 10.3390/plants13192699 PMC11478905

[37] Nakandala U, Furtado A, Henry RJ. Citrus genomes: past, present and future. Hortic Res. 2025;12(5):uhaf033. 10.1093/hr/uhaf033. 40224327 10.1093/hr/uhaf033 PMC11992330

[38] Tavaud M, Zanetto A, David JL, Laigret F, Dirlewanger E. Genetic relationships between diploid and allotetraploid cherry species (*Prunus avium*, *Prunus × gondouinii* and *Prunus cerasus*). Heredity. 2004;93(6):631–8. 10.1038/sj.hdy.6800589. 15354194 10.1038/sj.hdy.6800589

[39] Bharati R, Sen MK, Severová L, Svoboda R, Fernández-Cusimamani E. Polyploidization and genomic selection integration for grapevine breeding: a perspective. Front Plant Sci. 2023. 10.3389/fpls.2023.1248978. 38034577 10.3389/fpls.2023.1248978 PMC10684766

[40] Ferguson AR, Huang H. Cytology, ploidy and ploidy manipulation. In: Testolin R, Huang HW, Ferguson AR, editors. The Kiwifruit Genome. Cham: Springer International Publishing; 2016. p. 55–63. 10.1007/978-3-319-32274-2_5.

[41] Fiala J, Zezulová E, Nečas T. Evaluation of the genome size and ploidy level of pears (Pyrus spp.) in relation to their morphological traits. Horticulturae. 2024;10(12):1241. 10.3390/horticulturae10121241.

[42] Baccichet I, Foria S, Messina R, Peccol E, Losa A, Fabro M, et al. Genetic and ploidy diversity of pear (Pyrus spp.) germplasm of Friuli Venezia Giulia, Italy. Genet Resour Crop Evol. 2020;67(1):83–96. 10.1007/s10722-019-00856-9.

[43] Bielsa FJ, Irisarri P, Errea P, Pina A. Genetic diversity and structure of local pear cultivars from mountainous areas from Aragon (Northeastern Spain). Agronomy. 2021;11(9):1778. 10.3390/agronomy11091778.

[44] Irisarri P, Urrestarazu J, Ramos-Cabrer A, Pereira-Lorenzo S, Velázquez-Barrera ME, Díaz-Hernández MB, et al. Unlocking Spanish pear genetic diversity: strategies for construction of a national core collection. Sci Rep. 2024;14(1):26555. 10.1038/s41598-024-77532-1. 39489785 10.1038/s41598-024-77532-1 PMC11532559

[45] Brini W, Mars M, Hormaza JI. Genetic diversity in local Tunisian pears (*Pyrus communis* L.) studied with SSR markers. Sci Hortic. 2008;115(4):337–41. 10.1016/j.scienta.2007.10.012.

[46] Ouni R, Zborowska A, Sehic J, Choulak S, Hormaza JI, Garkava-Gustavsson L, et al. Genetic diversity and structure of tunisian local pear germplasm as revealed by SSR markers. Hortic Plant J. 2020;6(2):61–70. 10.1016/j.hpj.2020.03.003.

[47] Queiroz Á, Bagoin Guimarães J, Sánchez C, Simões F, Maia de Sousa R, Viegas W, et al. Genetic diversity and structure of the Portuguese pear (*Pyrus communis* L.) germplasm. Sustainability. 2019;11(19):5340. 10.3390/su11195340.

[48] HiDRAS website. Available from: https://sites.unimi.it/camelot/hidras/index.php. Accessed 4 July 2026.

[49] Vieira MLC, Santini L, Diniz AL, de Munhoz C. Microsatellite markers: what they mean and why they are so useful. Genet Mol Biol. 2016;39:312–28. 10.1590/1678-4685-GMB-2016-0027. 27561112 10.1590/1678-4685-GMB-2016-0027 PMC5004837

[50] Cipriani G, Marrazzo MT, Di Gaspero G, Pfeiffer A, Morgante M, Testolin R. A set of microsatellite markers with long core repeat optimized for grape (*Vitis* spp.) genotyping. BMC Plant Biol. 2008;8(1):127. 10.1186/1471-2229-8-127. 19087321 10.1186/1471-2229-8-127 PMC2625351

